# Interbrain Synchrony in the Expectation of Cooperation Behavior: A Hyperscanning Study Using Functional Near-Infrared Spectroscopy

**DOI:** 10.3389/fpsyg.2020.542093

**Published:** 2020-11-10

**Authors:** Mingming Zhang, Huibin Jia, Mengxue Zheng

**Affiliations:** ^1^Department of Psychology, College of Education, Shanghai Normal University, Shanghai, China; ^2^Department of Psychology, Henan University, Kaifeng, China; ^3^School of Teacher Education, Shaoxing University, Shaoxing, China; ^4^Faculty of Education, East China Normal University, Shanghai, China

**Keywords:** expectation of cooperation, hyperscanning, fNIRS, interbrain synchrony, sex effects

## Abstract

Expectation of others’ cooperative behavior plays a core role in economic cooperation. However, the dynamic neural substrates of expectation of cooperation (hereafter EOC) are little understood. To fully understand EOC behavior in more natural social interactions, the present study employed functional near-infrared spectroscopy (fNIRS) hyperscanning to simultaneously measure pairs of participants’ brain activations in a modified prisoner’s dilemma game (PDG). The data analysis revealed the following results. Firstly, under the high incentive condition, team EOC behavior elicited higher interbrain synchrony (IBS) in the right inferior frontal gyrus (rIFG) than individual EOC behavior. Meanwhile, the IBS in the IFG could predict the relationship between empathy/agreeableness and EOC behavior, and this prediction role was modulated by social environmental cues. These results indicate the involvement of the human mirror neuron system (MNS) in the EOC behavior and the different neural substrates between team EOC and individual EOC, which also conform with theory that social behavior was affected by internal (i.e., empathy/agreeableness) and external factors (i.e., incentive). Secondly, female dyads exhibited a higher IBS value of cooperative expectation than male dyads in the team EOC than the individual EOC in the dorsal medial prefrontal cortex (DMPFC), while in the individual EOC stage, the coherence value of female dyads was significantly higher than that of male dyads under the low incentive reward condition in the rIFG. These sex effects thus provide presumptive evidence that females are more sensitive to environmental cues and also suggest that during economic social interaction, females’ EOC behavior depends on more social cognitive abilities. Overall, these results raise intriguing questions for future research on human cooperative behaviors.

## Introduction

Expectation of cooperation (hereafter EOC) concerns how we think that another person is going to cooperate (Ng and Au, 2016). Pruitt and Kimmel proposed the “target/expectation theory” of cooperative behavior and highlighted the important role of EOC in establishing and promoting cooperative behavior (Pruitt and Kimmel, 1977; Sy et al., 2011). Previous research has postulated that an increasing trend in EOC enhances cooperation in general. However, EOC is not a sufficient condition for cooperation, as the positive effect of EOC on cooperation was moderated by individual differences and social environmental cues (Batson and Ahmad, 2001; Braver and Bongiolatti, 2002).

Bogaert et al. found that individuals with a pro-social social value orientation or higher trust toward others may expect that the other person is more likely to cooperate (Bogaert et al., 2011). Smeesters et al. (2003) reported that pro-social individuals expect more cooperation outcomes from their partners than do pro-selfs (Smeesters et al., 2003). The empathy–altruism hypothesis claims that empathy induction allows individuals to understand other people’s views and to imagine the feelings of others, which leads to an increase in individuals’ expectations of cooperation with others, and this expectation trend ultimately increases individuals’ willingness to cooperate (Kelley and Stahelski, 1970; Dawes et al., 1977). Recent empirical research shows that cooperative behavior in social dilemmas is only one kind of a more general class of behavior, namely, moral behavior, which includes reciprocity, respecting others’ property, honesty, equity, efficiency, as well as many others (Capraro and Perc, 2018). Meanwhile, some studies have reported that agreeableness predicts cooperation in different economic games (Ben-Ner et al., 2004; Pothos et al., 2011; Volk et al., 2011). It appears that agreeableness is positively associated with pro-social behavioral tendencies and at least accounts for some specific aspects of cooperation (Zettler et al., 2013). Similarly, this effect can also be extended to the sex effects modulating the relationship between EOC and cooperative outcome because females are generally considered pro-social and moral, while males are more likely to exhibit self-individual tendencies (Gregory et al., 2010; Lafko, 2015). Previous studies have also confirmed that females expect more cooperation behaviors from their partners than males (Bogaert et al., 2011).

Bogaert et al. (2011) declared that the relationship between EOC and cooperative behavior in social dilemmas is also moderated by social environmental cues (Bogaert et al., 2011). Accumulated researches have confirmed the modulation effect of social cues, e.g., Ng and Au (2016) found that game riskiness moderated the effect of EOC on cooperation such that the positive effect of EOC on cooperation was stronger for more risky games than for less risky games (Ng and Au, 2016). A similar finding is that people expect more cooperation when the payoff from mutual cooperation is higher (Charness et al., 2016). One plausible explanation is that a low incentive for mutual cooperation leads to higher risks of defection.

Beyond and based on experimental research, many mathematical and agent-based models have been presented to study cooperation in the social dilemma. These game theories reveal the essence of cooperation and competition: the ultimate goal is to maximize one’s own interests. From the earliest Nash equilibrium to the latest sub-game perfect equilibrium, these models have changed from static models to dynamic models, pure strategies to mixed strategies, and symmetric to asymmetric conditions. At the same time, it is also permeated and influenced by other methods and theories, e.g., Capraro and Perc (2018) studied the moral behavior with methods of statistical physics, which improved our understanding of the emergence of cooperation, also leading to new insights and contributing toward finding answers of cooperation and competition in social dilemma (Capraro and Perc, 2018). Meanwhile, the experimental research of social dilemmas and the establishment of models have gradually extended to the frame of multiple individuals and mixed strategies, e.g., in one economic exchange, *N* actors, relying on continuous production strategies and price strategies to participate *M* kinds of commodities (*N*, *M* > 1).

With the development of hyperscanning techniques, research on cooperative behavior has shifted from an experimental single-brain to a natural multi-brain framework (Hasson et al., 2012; Schilbach et al., 2013). Researchers have unraveled the underlying neural substrate of cooperative behavior in human–human interaction situations based on extensive behavioral researches that have clarified the involvement of cognitive control coupled with the mentalizing and mirror neuron networks in two-person cooperative behaviors. Thus, recent hyperscanning studies have revealed increased synchronized activity in the right superior frontal cortices and the medial prefrontal region across participants in cooperative actions (Funane et al., 2011; Cui et al., 2012; Dommer et al., 2012; Liu et al., 2016) and the right temporo-parietal junction in face-to-face economic cooperation (Tang et al., 2016) and synchrony of the anterior cingulate cortex and the prefrontal areas between the brains of paired subjects playing the prisoner’s dilemma game (PDG) (Astolfi et al., 2011). Moreover, team cooperative creativity studies have also confirmed increased inter-brain synchrony (IBS) in centralized mirror neuron networks and mentalizing systems (Lu et al., 2018; Xue et al., 2018; Mayseless et al., 2019). All these hyperscanning studies suggest that the mirror neuron networks and mentalizing systems are important for better cooperation and teamwork. It should be noted that several literatures have pointed an over-interpretation of the mirror neuron system (MNS) (Keysers, 2015). The present study follows the viewpoint of most researchers that MNS plays a part in social cognition.

Although much is known about the mechanism of team cooperative behavior based on several hyperscanning studies (Mayseless et al., 2019), little is known about the issue of team EOC behavior. Previous research indicates that EOC behavior involved the “social cognitive system” (together with the “reward system” and the “cognitive control system” forming the three psychological processes underlying social dilemma), which takes charge to process trust and threatening signals (e.g., mind reading) to urge people to decide in a social-orientation way (Van Lange et al., 2013), but these assumptions are based on the results of a single-brain framework study and the modulating effect of social environmental cues and individual differences on the relationship between EOC and cooperative behavior with their underlying neural substrate.

Since hyperscanning has promoted the study of social interaction behavior in more natural conditions and the core role of EOC in promoting social decision-making, in the present study, by setting up separate expectations stage and team co-expectations stage, the interpersonal neural mechanisms underlying the EOC behavior, especially co-expectation behavior, were analyzed using a hyperscanning technique of functional near-infrared spectroscopy (fNIRS). The participant dyads’ activations in the prefrontal and the bilateral inferior frontal regions, i.e., the regions of interest (ROIs), are measured simultaneously with the performance of a modified PDG.

Our goal and hypotheses for the present study were threefold: First, social environmental cues, individual differences (i.e., empathy traits, agreeableness, and gender in this study), and EOC behavior were assessed in order to reveal how they modulate the relationship between EOC behavior and cooperative outcome. These effects might yield distinct IBS patterns in related regions between conditions. Second, previous hyperscanning studies have shown significant differences between separate and team cooperative actions in two-person cooperative missions (Xu et al., 2012; Baker et al., 2016); thus, the participant dyads would show different IBS patterns across ROIs in these two separate stages, that is, the participants might show higher IBS in the co-expectation stage than in the separate expectation stage. Third, since some researches have reported gender effects in social interaction situations (Cheng et al., 2015; Zhang et al., 2017a,b), males and females might display different IBS patterns of EOC behavior in the present study.

## Materials and Methods

### Participants

Sixty-two healthy, right-handed university students (32 females and 30 males, mean age = 22.3 ± 2.4 years old, range 22–30 years) were recruited. All participated in pairs (31 pairs in total) with a partner of the same sex, and the participants in a dyad were unacquainted (strangers). The participants had normal or corrected-to-normal vision and were without psychiatric disorders or a psychiatric family history. Informed written consent was obtained from all the participants. The Southeast University Institutional Review Board approved all aspects of the experiments.

### Experimental Procedure

**FIGURE 1:**
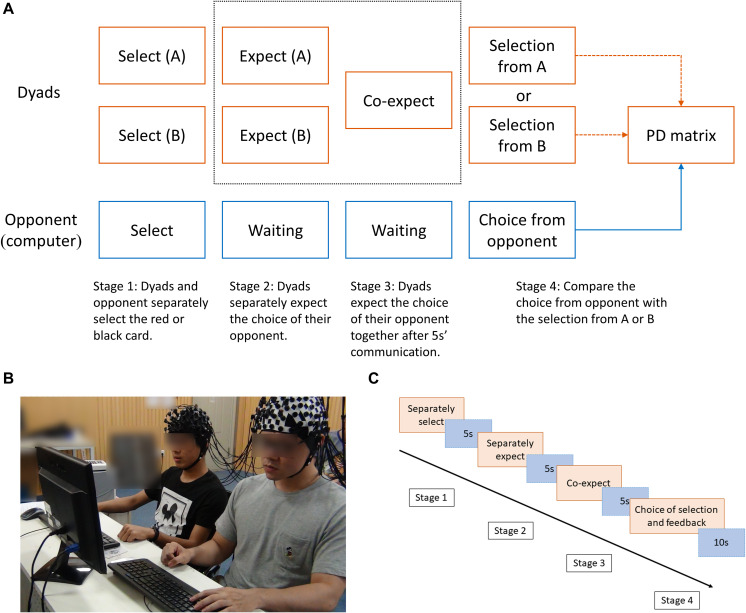
**(A)** Main experimental procedure. The gray dotted box represents the expectation of cooperation behavior. **(B)** Experimental setup. The dyads sitting side by side as cooperators. **(C)** Time process of one trial. The time process was divided into four periods (separate selection, separate expectation, co-expectation, and judging period).

The present study used an improved three-person PDG. Two participants sitting side by side acted as cooperators playing a computer-based PDG (see details in Figure 1). They were labeled as participants #A and #B. Prior to the experiment, the experimenter explained the rules. It should be noted that suggestive words like cooperation, non-cooperation, pro-social, or pro-self were never used in the instructions. The participants were given several practice rounds to familiarize themselves with the game and were prohibited from conversing verbally during the experiment. The participants were then asked to rest for 30 s, during which they were required to relax their minds and remain as motionless as possible (Jiang et al., 2015). The tasks were implemented using E-prime 2.0 (Psychology Software Tools, Inc., Pittsburgh, PA, United States).

The present PDG contained one selection stage and two expectation stages. First, the participants in the dyads had to choose a red or a black card, which formed selection scheme A and scheme B, and then expect their opponent’s (i.e., the computer) selection separately. Following a 5-s communication, they formed a mutual expectation. Finally, the computer uncovered its selection, which was a randomly chosen scheme (selection scheme A or B) in order to execute the prisoner’s dilemma matrix (Figure 1). The feedback in each round included the choice of selection scheme and the final judgment.

In a classic two-person PDG, if both players choose to cooperate, both receive the reward outcome (R). If one chooses to cooperate and one chooses to defect, the one who defected receives the temptation outcome (T), while the one who cooperated receives the sucker outcome (S). If both players choose to defect, both receive the punishment outcome (P) (Rapoport, 1967a). In the present study, there were two basic reward outcomes (R) for mutual cooperation: 3 yuan and 7 yuan (yuan is China’s currency), forming the low-incentive reward (hereafter LIR) conditions and the high-incentive reward (hereafter HIR) conditions. The two distinct trials (i.e., LIR and HIR) were performed in a random order. The temptation outcome was 10 yuan; the sucker outcome and the punishment outcome were 0 yuan (Table 1). The participants were told that their winnings would be given to them as remuneration after they completed the experiments, and their performance in the two expectation stages would also affect their remuneration. The monitor was used to present the stimuli, and keyboards were used to collect all the selection and expectation choices.

**TABLE 1:** The modified prisoner’s dilemma game matrix in the present study.

	**Cooperate (red)**	**Defect (black)**
Cooperate (red)	3/7	10
Defect (black)	10	0

*There were two basic reward outcomes for mutual cooperation: 3 yuan (low incentive reward) and 7 yuan (high incentive reward) and performed in a random order.*

The feedback options of the opponent (i.e., the computer) were controlled by a pre-configured E-prime program following the tit-for-tat strategy, whereby the opponent always makes the same choice that the two participants made in the previous trial (Sheldon, 1999; Van Lange and Visser, 1999). Previous research has reported that individualists often cooperate when confronted with a partner playing a tit-for-tat strategy because this would increase their personal benefits (Kuhlman and Marshello, 1975). Moreover, in order to ensure that the subjects could not detect this strategy in the present study, we added two types of interference feedback choices to confuse and mislead the subjects: (1) defect when the dyads choose to cooperate and (2) cooperate when dyads choose to defect. All the participants were interviewed after the experiment, and 87% of them (54 of the 62 subjects) believed that they were interacting with a real person.

The total experiment included 60 trials (30 trials for HIR and LIR, respectively), with each round lasting approximately 50 s. The Chinese version of the empathy questionnaire (empathy questionnaire for Chinese adults including 40 items on four-point scales) and the Big Five Questionnaire in Chinese version (BFQ) were collected from each participant after the test. By focusing on the separate and team co-expectation actions of the dyads, this modified paradigm allowed us to assess the behavioral and the neural difference of the EOC behavior. Meanwhile, the design setting of stranger dyads of the same sex and different incentive levels allowed the assessment of sex effects and modulation of social environmental cues.

### Apparatus

**FIGURE 2:**
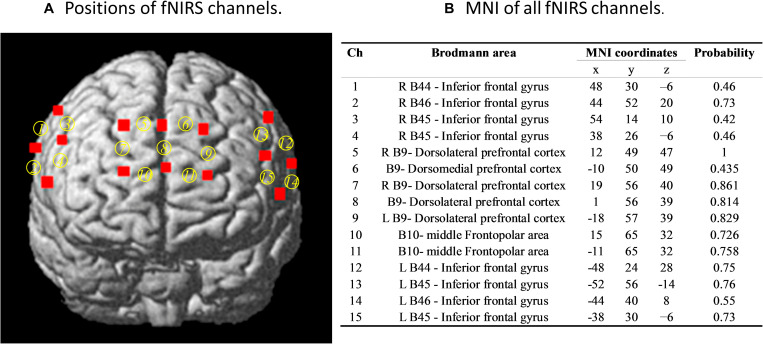
**(A)** Positions of functional near-infrared spectroscopy (fNIRS) channels and **(B)** the Montreal Neurological Institute (MNI) brain space of fNIRS channels. Six optodes (three emitters and three detectors) were attached to the forehead in a 2 × 3 lattice pattern, forming seven measurement channels, covering approximately the middle parts of the frontopolar area and the dorsal lateral prefrontal cortex. The remaining two optodes (two emitters and two detectors) were placed on the bilateral inferior frontal regions in two 2 × 2 lattice patterns forming, eight measurement channels. The probability here is that our measured MNI position covers the brain area.

We used a 30-channel fNIRS system (LABNIRS; Shimadzu Co., Japan) to simultaneously measure the concentration changes of oxygenated (oxy-Hb), deoxygenated (deoxy-Hb), and total hemoglobin (total-Hb) in the participants’ prefrontal and bilateral inferior frontal regions. For each participant in the dyad, one “3 × 3” and two “2 × 2” measurement patches were attached to a regular swimming cap with a 3-cm distance between one emitter and one detector, i.e., one channel, covering the prefrontal cortex (PFC) and bilateral inferior frontal gyrus (IFG), respectively. A 3D electromagnetic tracking device (FASTRAK; Polhemus, United States) was used to measure the precise positions of all fNIRS channels. The sampling rate was 42 Hz. The positions of all fNIRS channels and Montreal Neurological Institute brain space are reported in Figure 2.

### Data Analysis

We used the HOMER2 MATLAB package to remove longitudinal signal drift, motion artifact, and physiological noise, with the band-pass filter set to 0.01–0.1 Hz. HOMER2 is a set of MATLAB scripts used for analyzing fNIRS data to obtain estimates and maps of brain activation (see details in https://homer-fnirs.org/). After data preprocessing, the fNIRS data were further divided into four periods (separate selection, separate expectation, co-expectation, and judging period) according to the experimental design. We mainly focused on two expectation stages and the judging period in the present study. In the CE (short for co-expectation) stage, choices from dyads were classified into cooperative expectation (i.e., expecting the computer to choose the red card) and defective expectation (i.e., expecting the computer to choose the black card). In the SE (short for separate expectation) stage, the definition of the dyads’ expectation choices was in line with those of the CE stage, except that the roles of the two members in a pair might differ. Note that the dyad members might make different choices in the SE stage. This situation is not involved in the present study (e.g., one expects red, while the other expects black) (see the details in Table 2).

**TABLE 2:** Choices by dyads in the two expectation stages.

	**Separate expectation**		**Co-expectation**
	**Sub #A**	**Sub #B**	
Expectation choices	Red	Red	Red or black
	Red	Black	
	Black	Red	
	Black	Black	

*Expectation choices were classified into cooperative expectation (expecting the computer to choose the red card) and defective expectation (expecting the computer to choose the black card).*

**FIGURE 3:**
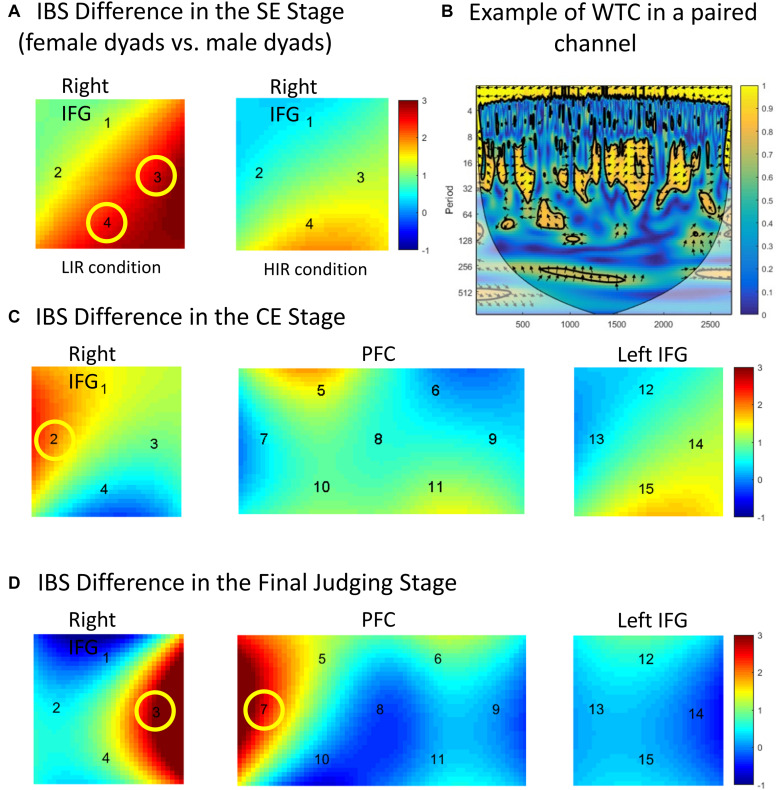
**(A)** Interbrain synchrony (IBS) differences in the separate expectation stage. Female dyads evoked higher IBS than male dyads in the right inferior frontal gyrus (IFG) (CH 3 and 4) under the low incentive reward (LIR) condition. **(B)** Example of wavelet coherence. The data were from two participants’ the same channel (CH 1). **(C)** IBS differences in the co-expectation stage. IBS under the high incentive reward condition was higher than that under the LIR condition in the right IFG (CH 2). **(D)** IBS differences in the final judging stage. Successful team co-expectation yielded higher IBS than unsuccessful team co-expectation in the right IFG (CH 3) and the dorsal lateral prefrontal cortex (CH 7). Colored bars indicate *t*-values.

To examine the inter-brain coupling between the dyads, we used the wavelet coherence MATLAB package to calculate the wavelet coherence (WTC) in order to quantify the inter-brain synchrony of each dyad. Wavelet coherence was used to measure the cross-correlations between time series. Compared with traditional correlation methods, wavelet coherence measures the correlation between two signals’ components on both frequency and time domains. Moreover, it is more capable of uncovering locally phase-locked behavior than the Fourier analysis (Grinsted et al., 2004). WTC has been used successfully in previous fNIRS hyperscanning studies (Cui et al., 2012; Cheng et al., 2015; Zhang et al., 2017a,b). In the present study, we obtained WTC in each event and averaged them. In order to remove the high- and low-frequency noises, such as those associated with respiration (about 0.2–0.3 Hz) and cardiac pulsation (about 1 Hz), frequency period of 5–100 s (corresponding to frequency 0.01–0.2 Hz, respectively) was selected for statistical analyses (see the example in Figure 3B). Note that we primarily focused on the oxy-Hb data since the oxygenated signal was more sensitive to changes in cerebral blood flow (Hoshi, 2003; Lindenberger et al., 2009).

## Statistical Test and Results

### Behavioral Data

#### Individual Differences

The participants’ empathy was assessed with a four-scale (1: strongly disagree to 4: strongly agree) questionnaire. We also extracted the agreeableness score from the BFQ. The empathy score and the agreeableness score of each dyad were obtained *via* averaging the scores of the two participants within each dyad. To examine the effect of sex and task type on individual trait scores (empathy and agreeableness), a two-way ANOVA [sex (male vs. female) × task type (LIR vs. HIR)] was conducted on the empathy score and agreeableness score, respectively, from all the dyads. As expected, the results did not reveal a significant main effect of task type, sex, and interaction effect (*P* > 0.05). Pearson correlation analyses were conducted to calculate the relationship between agreeableness and empathy scores. The results indicated that the agreeableness and the empathy scores were positively correlated (*r* = 0.373, *p* = 0.011, two-tailed).

#### Reaction Times and Reaction Choices

In the SE stage, in order to examine the effects of sex, task type, and expectation type on the reaction times (RTs) and reaction choices (i.e., the number of each kind of selection scheme from the participants), three-factor repeated-measures ANOVA [sex (male vs. female) × task type (LIR vs. HIR) × expectation type (cooperation vs. defection)] was conducted on the RTs and reaction choices of all the dyads. The RT of each dyad was obtained *via* averaging the RTs of the two participants within each dyad. For the RTs, there was a significant main effect for expectation types (cooperation vs. defection) [*F*(1, 29) = 9.156, *p* = 0.003, η*_*p*_*^2^ = 0.269; false discovery rate (FDR)-corrected], and the *post hoc* test revealed that the average reaction time of cooperative expectation (*M* = 2,141.57 ms, *SD* = 1,824.08) was shorter than that of defective expectation (*M* = 2,449.66 ms, *SD* = 2,118.70). No significant effect was found for the reaction choices.

In the CE stage, a similar three-factor repeated-measures ANOVA [sex (male vs. female) × task type (LIR vs. HIR) × expectation type (cooperation vs. defection)] was conducted. For the RTs, there was a significant interaction effect between expectation type and task type [*F*(1, 29) = 6.670, *p* = 0.010, η*_*p*_*^2^ = 0.030 (FDR-corrected)]. A simple effect analysis revealed that, under the HIR condition, the dyads formed cooperative expectations (*M* = 1,857.47 ms, *SD* = 2,722.262) faster than defective expectations (*M* = 2,043.06 ms, *SD* = 1,896.799). For the reaction choices, there was a significant interaction effect between task type and expectation type [*F*(1, 29) = 3.470, *p* = 0.033, η*_*p*_*^2^ = 0.107 (FDR-corrected)]. A simple effect analysis revealed that, under the HIR condition, the dyads tended to make more cooperative expectations (*M* = 21.66, *SD* = 3.93) than defective expectations (*M* = 8.34, *SD* = 3.93).

### Interbrain Synchrony

In the SE stage, the fact that the dyad members usually make different expectations (e.g., one expects red, the other expects black) could not make us analyze the effect of expected type (cooperation vs. defection) on IBS quantified by WTC. Thus, a two-factor repeated-measures ANOVA [sex (male vs. female) × task-type (LIR vs. HIR)] was conducted of the coherence values of all scalp channels from all the dyads. The IBS increase was defined as a higher average coherence value. There was no main effect of sex or task type on all the channels (*P* > 0.05). There was a significant interaction effect between task type and sex in the right IFG [CH 3: *F*(1, 29) = 8.673, *p* = 0.009, η*_*p*_*^2^ = 0.301; CH 4: *F*(1, 29) = 9.184, *p* = 0.002, η*_*p*_*^2^ = 0.317 (FDR-corrected)]. A simple effect analysis revealed that, under the LIR condition, the female dyads evoked a higher IBS than the male dyads in the right IFG (CH 3: *p* = 0.012; CH 4: *p* = 0.004) (see the details in Figure 3A).

In the CE stages, a three-factor repeated-measures ANOVA [sex (male vs. female) × expectation type (cooperation vs. defection) × task type (LIR vs. HIR)] was conducted on all the channels. In the right IFG (CH 2), there was a significant main effect of task type [*F*(1, 29) = 12.860, *p* = 0.001, η*_*p*_*^2^ = 0.331 (FDR-corrected)]. *Post hoc* tests revealed a significantly greater coherence under the HIR condition than under the LIR condition (*p* = 0.001). There was no other significant main effect and interaction effect (*P* > 0.05) (see the details in Figure 3C).

In the final judging stages, the IBS evoked by expectation results (i.e., dyads’ co-expectation correctly predicts their opponent’s choice or not) was analyzed. Three-factor repeated-measures ANOVA [sex (male vs. female) × expectation result (successful expectation vs. unsuccessful expectation) × task type (LIR vs. HIR)] was conducted in the coherence values of all the channels from all the dyads. There was a significant main effect in expectation result in the right IFG [CH 3: *F*(1, 29) = 7.158, *p* = 0.012, η*_*p*_*^2^ = 0.224 (FDR-corrected)] and the dorsal lateral prefrontal cortex (DLPFC) [CH 7: *F*(1, 29) = 10.836, *p* = 0.001, η*_*p*_*^2^ = 0.307 (FDR-corrected)]. *Post hoc* tests revealed that there was a significant coherence increase in the right IFG and the DLPFC if the dyads formed a successful co-expectation (successfully expect the choice of their opponent) (CH 3: *p* = 0.012; CH 7: *p* = 0.001). There was no main effect of sex or task type (*P* > 0.05) (see the details in Figure 3D).

In order to test the effects of sex and stage type (SE vs. CE vs. baseline vs. judging stages) on IBS, a two-factor repeated-measures ANOVA [sex (male vs. female) × stage type (SE vs. CE vs. baseline vs. judging stages)] was conducted of the coherence values from all the dyads. There was a significant main effect of stage type in the rIFG [CH 2: *F*(1, 29) = 9.064, *p* = 0.003, η*_*p*_*^2^ = 0.284 (FDR-corrected); CH 3: *F*(1, 29) = 7.268, *p* = 0.011, η*_*p*_*^2^ = 0.237 (FDR-corrected)] and the middle frontopolar area [CH 10: *F*(1, 29) = 11.708, *p* < 0.001, η*_*p*_*^2^ = 0.325 (FDR-corrected)]. *Post hoc* tests revealed that the IBS in the SE, CE, and judging stages were significantly higher than the baseline in the rIFG [SE (CH 2: *p* = 0.023); CE (CH 2: *p* = 0.012; CH 3: *p* = 0.008); judging stage (CH 2: *p* = 0.015)], and the IBS in the SE and CE stages were significantly higher than the baseline in the middle frontopolar area [SE (CH 10: *p* = 0.012); CE (CH 10: *p* = 0.007)]. There was a significant interaction effect between stage type and sex in the DMPFC [CH 6: *F*(1, 29) = 8.136, *p* = 0.008, η*_*p*_*^2^ = 0.247 (FDR-corrected)]. A simple effect analysis revealed that there was a significant coherence increase in the CE stage over the SE stage in female dyads (*p* = 0.010), but not in male dyads (*p* = 0.390). There were no significant main effects (*P* > 0.05).

### The Neural–Behavior Relationship

To assess the relationship between the dyads’ individual differences and IBS, a Pearson correlation analysis was conducted to calculate the relationship between IBS values and empathy/agreeableness scores. We regarded the individual differences as a coupled unit, and the mean scores of the dyads’ empathy and agreeableness scores were calculated.

In the SE stage, when the dyads formed cooperative expectations under the HIR condition, IBS and agreeableness were positively correlated in the right IFG (CH 4: *r* = 0.653, *p* = 0.009; CH 15: *r* = 0.546, *p* = 0.020).

In the CE stage, when the dyads formed cooperative expectations under the HIR condition, IBS and agreeableness/empathy were positively correlated in the IFG [empathy (CH 2: *r* = 0.536, *p* = 0.021 and CH4: *r* = 0.514, *p* = 0.024); agreeableness (CH 1: *r* = 0.634, *p* = 0.010; CH 3: *r* = 0.675, *p* = 0.004; CH 4: *r* = 0.537, *p* = 0.021; CH14: *r* = 0.663, *p* = 0.007)]. When the dyads formed defective expectations, IBS and empathy were negatively correlated in the right IFG (CH 2: *r* = −0.523, *p* = 0.021). This relationship was not significant in other brain cortexes and conditions (*P* > 0.05) (see the details in Table 3; only significant results are reported).

**TABLE 3:** The neural–behavioral relation in all conditions.

**Stage**	**Task**	**Expectation**	**CH**	**Region of interest**	**Individual differences—interbrain synchrony**	
					**Empathy**	**Agreeableness**
Separate expectation	Low incentive reward	Cooperation	–	–	–	–
		Defection	–	–	–	–
	High incentive reward	Cooperation	(4)	Right inferior frontal gyrus	–	0.653**
			(15)	Left inferior frontal gyrus	–	0.546*
		Defection	–	–	–	–
Co-expectation	Low incentive reward	Cooperation	–	–	–	–
		Defection	–	–	–	–
	High incentive reward	Cooperation	(1)	Right inferior frontal gyrus	–	0.634**
			(2)	Right inferior frontal gyrus	0.536*	–
			(3)	Right inferior frontal gyrus	–	0.675**
			(4)	Right inferior frontal gyrus	0.514*	0.537*
			(14)	Left inferior frontal gyrus	–	0.663**
		Defection	(2)	Right inferior frontal gyrus	−0.523*	–

*The number inside the parentheses represents the number of the dyads who showed significant correlation based on the correlation analysis.* * *p < 0.05.* ** *p < 0.01.*

## Discussion

In the present study, we used an fNIRS hyperscanning system to simultaneously measure the pair of participants’ IBS in an iterated modified PDG to investigate the EOC behavior. To the best of our knowledge, the present study is the first such attempt to investigate the underlying substrate of inter-brain synchrony of the EOC behavior in human-to-human interaction.

Our behavioral and inter-brain results confirmed the initial hypothesis regarding the mediating effect of individual differences, social cues, and sex. The behavioral results demonstrated that cooperative expectation was a common tactic across all conditions of the present study, i.e., higher rates in the CE stage and shorter reaction times in the SE stage. Moreover, the incentive level modulated the EOC behavior, i.e., more cooperative expectations and shorter reaction times under the HIR condition. A previous study found that a person may also expect the other players to be more likely to cooperate in larger incentive games, showing that HIR is more conducive to cooperation (Rapoport, 1967b). However, this modulation was only found in the CE period. One possible interpretation is that the mutual communication (in the CE stage) promoted cooperative expectation under the HIR condition, yet the “fear” of being defected by their opponent (pursuing the temptation outcome) reduced the likelihood of cooperative behavior under the LIR condition. However, the results did not reveal differences between male and female dyads in all conditions. This was consistent with the behavioral findings of previous hyperscanning studies, especially in the study of decision-making behaviors (Zhang et al., 2017a).

### Social Environmental Cues Evoked Differences in IBS Performance

Concerning the IBS of EOC, although there was no significant difference between incentive levels in the behavioral data, the inter-brain analysis showed significant findings. i.e., the participant pairs showed an increase in IBS value under the HIR condition than that of the LIR condition in the right IFG (CE stage). Concerning interpersonal interactions, previous studies have demonstrated that the MNS, mainly including the IFG and the inferior parietal lobule, enables an individual to understand others’ actions and intentions *via* embodied simulation (Iacoboni, 2008; Liu et al., 2017). Numerous studies have shown that interaction in synchrony with other persons fosters the IBS in the IFG, e.g., Koike et al. (2015) have examined the neural substrates of shared attention in a real-time mutual gaze task and demonstrated IBS in the right IFG (Koike et al., 2015). Mayseless et al. examined creative problem-solving involving team cooperation in a naturalistic study design and found an increased IBS for cooperation in the left IFG (Mayseless et al., 2019). With respect to the higher IBS values in the IFG under the HIR condition in the present study, combined with the behavioral results, one plausible explanation is that higher incentives induce common goals and less self-other distinction, and it is thus relatively simple to achieve a mutual understanding of actions and intentions (Liu et al., 2015). In general, the present findings revealed a modulation effect of external environmental cue (i.e., incentive levels) in the inter-brain networks. At the same time, the modulation effect of external environmental cues was absent in the SE stage, that is, there was no significant IBS difference between task type (HIR/LIR) in the SE stage. Considering the behavioral results (no significant difference between task type in RTs and reaction choices), one reasonable explanation is that, in indirect social interaction situations (respective action without direct interaction), the dyad members expect separately and lack communication. This resulted in the absence of interbrain synchrony across task types. This finding also provided a new neural indicator (i.e., IBS) and underlying neural substrate between individual EOC behavior and team EOC behavior.

Moreover, in the final section stage, successful expectation elicited higher IBS than unsuccessful expectation in the right IFG and the DLPFC. Previous studies have shown that the right DLPFC is activated in moral decisions and involved in a more “cognitive” subsystem that elicits utilitarian reasoning (Sanfey et al., 2003). Liu et al. (2012) investigated the neural mechanism of intertemporal choice and found that the IFG and the DLPFC was active in a reward-based model (Liu et al., 2012). Thus, it is not difficult to understand the findings in the present study, that is, in the final section stage, compared with unsuccessful expectation, successful expectation seems to be an affirmation and self-reward to dyads. Meanwhile, as described in the “Materials and Methods” sections, the performance of the expectation directly affects their remuneration. This reward stimuli leads to the synchronous activation in the right IFG and the DLPFC of the dyads.

Similarly, the significant differences of IBS between the task states (SE, CE, and judging stages) and the resting stage (i.e., baseline) indicated the successfully experimental paradigm and the involvement of MNS in the EOC behavior in a social interaction context. Meanwhile, the IBS of the SE and the CE stages are significantly higher than the resting stages in the Bradman 10 area (the middle frontopolar area). Based on previous research on the relationship between neural substrate and social cognition, a significant activation of this region may be related to multitasking (i.e., advanced cognitive retrieval) and mentalizing (Okamoto et al., 2004; Maidan et al., 2015). This area has also been proven responsible for playing a role in promoting cognitive and mentalizing abilities in a two-person decision-making task (Balconi et al., 2017). In the present study, we believe that the expectation behavior yielded the synchronous activation in the Bradman 10 area. This needs to be confirmed by future research.

## IBS in the IFG Predict the Relationship Between Empathy/Agreeableness and EOC Behavior

The neural–behavioral results also suggest a prediction role of IBS in the relationship between empathy/agreeableness and EOC behavior. First, the IBS of the IFG in relationships between trait of empathy and outcomes of the EOC behavior was demonstrated only under HIR task in the CE stage, but not under LIR task in the SE stage. As discussed above, a higher incentive involves common goals and less self-other distinction, making it relatively simple to achieve a mutual understanding of actions and intentions (Liu et al., 2015). Meanwhile, concerning the lower involvement of empathy in the SE stage, the absence of a prediction role of IBS under LIR task in the SE stage is not difficult to understand. Our research showed that empathy could predict EOC behavior at least in the inter-brain level, which strongly complements the modulation effect of empathy on cooperative behavior.

Otherwise, the IBS of the IFG in relationships between agreeableness and the outcomes of the EOC behavior was demonstrated only in the HIR task, but not in the LIR task. Previous research has suggested that agreeableness accounts for some specific aspects of cooperation (Zettler et al., 2013). Regarding the relationship between personality and behavior, it is generally accepted that personality traits, with environmental factors, jointly determine the individual’s behavior (Magnusson, 1990). Meanwhile, a previous hyperscanning study has shown that the participants’ empathy was significantly correlated with their IBS values in the bilateral IFG (Liu et al., 2017). The results of the present study show that EOC behavior is also regulated by both personality traits and environmental factors. This finding extends not only to the neural indicators (i.e., IBS) but also the new content (i.e., EOC behavior) to the study of the relationship between personality and behavior.

### Social Environmental Cues Modulate Sex Effects in the Two Expectation Stages

With respect to the sex effect, the social environmental cues modulate an IBS difference between sex in the SE stage; the coherence value of the female dyads was significantly higher than that of the male dyads under the LIR condition in the rIFG. As described above, the rIFG enables an individual to understand others’ actions and intentions (Iacoboni, 2008; Liu et al., 2017). Thus, this result indicates that females are more sensitive to their partners in indirect social interaction situations. Previous research has demonstrated that, during economic social interactions, males may primarily depend on non-social cognitive abilities to make risky decisions in a social interaction, while females may use both social and non-social cognitive abilities (Zhang et al., 2017a). Our interpretation of the sex effect found in the right IFG is that females were more sensitive to the social environmental cue (incentive level), resulting in a higher IBS value than in males during the SE stage. This might also support the evolutionary biological perspective that females are more sensitive to imperceptible changes (Hyde, 2005). A previous fMRI study found that social interactions evoke four to seven brain areas in males but as many as 14–16 brain areas for interpreting meaning, tone, and body language in females (Balliet et al., 2011).

Furthermore, among the most interesting findings in this study is the sex effect between individual expectation (SE stage) and co-expectation (in the CE stage) in the DMPFC. For the interpretation of this result, two closely optimal explanations may make sense. Concerning the impact of social cues and the less involvement of indirect interaction in the SE stage, a higher IBS of cooperative expectation in the CE stage is not hard to understand. The alternative possibility is that the IBS value increase in the DMPFC might indicate the pro-social effect, which refers to a phenomenon whereby people tend to be more pro-social after synchronizing behaviors with others (Reddish et al., 2013; Endedijk et al., 2015). Previous studies have shown that synchronously moving with others (e.g., walking, singing, and tapping) fosters pro-sociality (Wiltermuth and Heath, 2009; Kirschner and Tomasello, 2010; Cirelli et al., 2014; Koehne et al., 2016). In a multi-brain frame research, Hu et al. (2017) studied the mutual pro-sociality effect using a simultaneous key pressing task after silent time-counting and found IBS in the left middle frontal cortex (Hu et al., 2017). In our work, the female dyads showed a stronger cooperative tendency in neural network (DMPFC) after a short synchronous interaction (i.e., co-expectation behavior). To some extent, our results complement the neural information of mutual pro-sociality effect. Moreover, the DMPFC is thought to be part of the theory-of -mind brain networks, activated by considering the intentions of another individual in social processing (Isoda and Noritake, 2013). Rilling et al. (2004) reported that partner feedback in the PDG reliably activates the DMPFC, and this activation is engaged more when playing with a human than a computer partner (Rilling et al., 2004), indicating that females are sensitive to feedback even when interacting with a computer opponent. These results are also supported by the “theory of social representations,” which posits that individuals’ social behavior is controlled by their inherent “representations system,” and this “representations system” is affected by certain social factors such as culture and education. According to the “theory of social representations,” the sex effect in the current study demonstrated that males and females execute different “representations systems” during economic social interaction, while females’ EOC behavior depends on more social cognitive abilities.

### The Effect of Group Size and Possible Applications in the Future

The EOC behavior is inevitably affected by the size of the group, which is the decisive factor that determines whether the spontaneous and rational pursuit of individual interests will lead to a favorable group behavior (Olson, 1971). A recent study has shown that the deficits scaled approximately linear with group size; the negative trends tended to accelerate a little faster downward for larger groups (Wang et al., 2020). Olson declared that the more people who share the benefits and the lesser the individuals who carry out activities for the realization of the group benefit, economic or rational individuals will not act for the common interests of the group (Olson, 1971). However, most of the conclusions and hypotheses are based on previous studies drawn from the public goods game, which combined the conflicts between individual interests and group interests. In the present research, we pay more attention to the group behavior that align individual interests with collective interests. Therefore, we have to be aware of the doubt if the EOC behaviors also conform to the rule of group size, the latter needing to be confirmed by future studies.

There is, in addition, one further point to make. For the purpose of application and practice, the research outcome of the EOC behavior and social dilemma should also apply to future research and daily life. A very recent research shows that communicating sentiment may also increase cooperation, which in turn can lead to better climate agreement—a very well-known social dilemma (Wang et al., 2020). The study of EOC behavior promotes the understanding of neuroeconomic research. On the other hand, the application of game theory in daily life facilitates the solution of economic and even global problems, for example, using the game theory to study and solve the problem of water resources allocation (Wang et al., 2003) and global climate change (Wang et al., 2020). In the solution of these practical and global problems, the in-depth study of EOC behavior will make important contributions.

Finally, the present study also comes with limitations and further questions for future research. First, it should be noted that the sex effects found for the Chinese sample are highly consistent with sex stereotypes in the Chinese culture, where females traditionally have been expected to be more neurotic and tender-minded than males. These sex effects in previous cooperative behaviors might differ across cultures, e.g., Cheng et al. (2015) and Baker et al. (2016) both used the computer-based cooperation task to study cooperative behavior, notably finding completely different results of significant IBS (Cheng et al., 2015; Baker et al., 2016). An intriguing possibility is that cultural differences between study populations drawn from predominantly Asian vs. Western societies lead to different patterns of inter-brain coherence during cooperation (Baker et al., 2016). In addition, our participants were concentrated among college students, which may present some kind of personality homogeneity. Previous studies have confirmed that individuals can present the heterogeneity, which may help to promote the expectation of cooperation (Li et al., 2019a,b). Therefore, whether the results of this study can be generalized to a wider range of people also needs further confirmation in future studies. Second, the fact that computer opponents are able to activate the network, albeit to a lesser extent than human opponents, suggests that this neural system can also be activated by reasoning about the unobservable states of non-human systems. However, we still suggest further research to consider human opponents as a new orientation. Finally, as the sample size was relatively small, further empirical testing is needed to confirm the present findings, especially regarding the sex effects.

## Conclusion

The present study concludes with three main findings. First, HIR condition showed higher IBS values than LIR condition in the IFG, which might reveal a modulation effect of external environmental cue (i.e., incentive levels) in the inter-brain networks. Second, IBS in the IFG predicts the relationship between empathy/agreeableness and EOC behavior. This finding strongly complements the modulation effect of empathy on cooperative behavior and provides new neural indicators (i.e., IBS) and new content (i.e., EOC behavior) to the study of the relationship between personality and behavior. Third, there was a sex effect between team and individual EOC behavior in the DMPFC, and in the SE stage, the coherence value of the female dyads was significantly higher than the male dyads under the LIR condition in the rIFG. This sex effect thus provides a presumptive evidence supporting the evolutionary biological perspective that females are more sensitive to imperceptible changes in neurological levels as well as that, during economic social interaction, females’ EOC behavior may depend on more social cognitive abilities. These results suggest that males and females may have different “representations systems” in the processing of EOC behavior and also indicate a pro-social effect in female dyads. Overall, this research on EOC behavior in the human-to-human interactions raises intriguing questions for future research.

## Data Availability Statement

The raw data supporting the conclusions of this article will be made available by the authors, without undue reservation.

## Ethics Statement

The studies involving human participants were reviewed and approved by the Southeast University Institutional Review Board. The patients/participants provided their written informed consent to participate in this study. Written informed consent was obtained from the individual(s) for the publication of any potentially identifiable images or data included in this article.

## Author Contributions

MiZ and MeZ designed the research and wrote the manuscript. MiZ and HJ performed the research. MiZ analyzed the data. All authors contributed to the article and approved the submitted version.

## Conflict of Interest

The authors declare that the research was conducted in the absence of any commercial or financial relationships that could be construed as a potential conflict of interest.
