# Supplementary material for: Interbrain Synchrony in the Expectation of Cooperation Behavior: A Hyperscanning Study Using Functional Near-Infrared Spectroscopy
Source: Front Psychol. 2020 Nov 10;11:542093. doi: 10.3389/fpsyg.2020.542093 (PMC7721002; doi:10.3389/fpsyg.2020.542093)
Supplement: Supplementary file 1 [file Data_Sheet_1.PDF]

Supplementary Materials

Table 1

Table 1 Reaction Times (RTs) in the SE and CE stages

Stage	Sex	Task	Expectation type	(Task/Sex)	(Task/ Expectation type)	(Expectation type/Sex)	(Task/Sex/ Expectation type)
SE	Male: M = 2265.73, SD = 2161.09	LIR: M = 2440.77 SD = 1968.51	Cooperation: M = 2141.57, SD = 1824.08	Male/LIR: M = 2265.29, SD = 1922.17	LIR/Cooperation: M = 2089.73, SD = 2263.88	Male/Cooperation: M = 2139.98, SD = 2303.44	Male/LIR/Cooperation: M = 2316.15, SD = 1949.06
							Male/LIR/ Defection: M = 2214.43, SD = 2095.28
				Male/HIR: M = 2266.17, SD = 1790.01	LIR/Defection: M = 2491.80, SD = 2353.13	Male/ Defection: M = 2391.48, SD = 1418.73	Male/HIR/Cooperation: M = 2198.10, SD = 2102.89
							Male/HIR/ Defection: M = 2334.24, SD = 1797.13
	Female: M =2325.5, SD =2161.09	HIR: M = 2200.47 SD = 1874.28	Defection: M = 2449.66, SD = 2118.70	Female/LIR: M = 2377.91, SD = 1909.18	HIR/Cooperation: M = 2193.41, SD = 2084.28	Female/Cooperation: M = 2143.16, SD = 1344.72	Female/LIR/Cooperation: M = 2378.66, SD = 1876.02
							Female /LIR/ Defection: M = 2377.16, SD = 1642.34
				Female/HIR: M = 2273.09, SD = 2093.10	HIR/Defection: M = 2507.52, SD = 1664.27	Female/Defection: M = 2507.84, SD = 1818.67	Female /HIR/Cooperation: M = 2232.79, SD = 1358.43
							Female /HIR/ Defection: M = 2313.39, SD = 1667.57
CE	Male: M =1931.52, SD =2013.37	LIR: M =1867.32, SD =1537.17	Cooperation: M = 1824.62, SD = 1415.44	Male/LIR: M = 1844.71, SD = 1994.55	LIR/Cooperation: M = 1791.76, SD = 2108.61	Male/Cooperation: M=1890.61, SD=1751.75	Male/LIR/Cooperation: M = 1905.31, SD = 2187.12
							Male/LIR/ Defection: M = 1784.11, SD = 2401.98
				Male/HIR: M = 2018.33, SD = 1832.18	LIR/Defection: M = 1942.88, SD = 1765.73	Male/Defection: M = 2072.43, SD = 1994.99	Male/HIR/Cooperation: M = 1978.27, SD = 1934.27
							Male/HIR/ Defection: M = 2058.39, SD = 2130.09

	Female: M = 1886.06, SD = 1733.23	HIR: M = 1950.26, SD = 1609.53	Defection: M = 1992.97, SD = 2031.26	Female/LIR: M = 1889.93, SD = 1779.79	HIR/Cooperation: M = 1857.47, SD = 1222.26	Female/Cooperation: M = 1758.63, SD = 1499.13	Female/LIR/Cooperation: M = 1871.11, SD = 1485.63
							Female /LIR/ Defection: M = 1902.75, SD = 1473.95
				Female/HIR: M = 1882.19, SD = 1386.38	HIR/Defection: M = 2043.06, SD = 1496.79	Female/ Defection: M = 1913.51, SD = 1367.53	Female /HIR/Cooperation: M = 1927.44, SD = 1298.01
							Female /HIR/ Defection: M = 1836.94, SD = 1175.74

Table 2

Table 2 Reaction Choices in the SE and CE stages

Stage	(Task/ Expectation type)	(Expectation type/Sex)	(Task/Sex/Expectation type)
SE	LIR/Cooperation: M = 13.26, SD = 3.11	Male/Cooperation: M = 26.74, SD = 4.02	Male/LIR/Cooperation: M = 12.07, SD = 5.99
			Male/LIR/ Defection: M = 17.93, SD = 5.99
	LIR/Defection: M = 16.74, SD =3.11	Male/ Defection: M = 33.26, SD = 4.02	Female/LIR/Cooperation: M = 14.45, SD = 4.43
			Female /LIR/ Defection: M = 15.55, SD = 4.43
	HIR/Cooperation: M = 16.10, SD = 2.36	Female/Cooperation: M = 31.99, SD = 2.67	Male/HIR/Cooperation: M = 14.67, SD = 2.16
			Male/HIR/ Defection: M = 15.33, SD = 2.16
	HIR/Defection: M = 13.90, SD = 2.36	Female/Defection: M = 28.01, SD = 2.67	Female /HIR/Cooperation: M = 17.54, SD = 2.35
			Female /HIR/ Defection: M = 12.46, SD = 2.35

CE	LIR/Cooperation: M = 13.39, SD = 4.16	Male/Cooperation: M = 35.63, SD = 5.93	Male/LIR/Cooperation: M = 14.97, SD = 3.37
			Male/LIR/ Defection: M = 15.03, SD = 3.37
	LIR/Defection: M = 16.61, SD = 4.16	Male/ Defection: M = 25.37, SD = 5.93	Female/LIR/Cooperation: M = 11.81, SD = 4.01
			Female /LIR/ Defection: M = 18.19, SD = 4.01
	HIR/Cooperation: M = 21.66, SD = 3.93	Female/Cooperation: M = 34.47, SD = 4.67	Male/HIR/Cooperation: M = 20.66, SD = 4.58
			Male/HIR/ Defection: M = 10.34, SD = 4.58
	HIR/Defection: M = 8.34, SD = 3.93	Female/Defection: M = 25.53, SD = 4.67	Female /HIR/Cooperation: M = 22.66, SD = 2.77
			Female /HIR/ Defection: M = 6.34, SD = 2.77

Table 3

Table 3 Result of the two-factor repeated measures ANOVA in the SE stage.

Ch	Main effect of task-type		Main effect of sex		Interaction effect	
	F	p	F	p	F	p
1	0.154	0.699	0.819	0.376	0.769	0.390
2	1.856	0.187	1.897	0.177	3.412	0.051
3	3.623	0.079	3.476	0.081	8.673	0.009
4	4.323	0.050	0.055	0.816	9.184	0.002
5	0.023	0.880	0.769	0.390	0.211	0.650

6	0.154	0.699	0.234	0.634	0.425	0.522
7	0.023	0.880	0.044	0.837	0.318	0.579
8	0.425	0.522	0.455	0.507	0.547	0.468
9	0.318	0.579	2.663	0.118	2.164	0.156
10	0.722	0.405	0.518	0.480	0.722	0.405
11	2.671	0.108	0.269	0.609	1.301	0.267
12	1.109	0.253	0.236	0.632	0.900	0.353
13	2.623	0.120	1.241	0.219	1.400	0.250
14	2.219	0.139	1.055	0.27	0.126	0.759
15	2.625	0.112	0.683	0.418	0.962	0.112

Table 4

Table 4 Result of the three-factor repeated measures ANOVA in the CE stage.

Ch	Main effect of task		Main effect of sex		Main effect of expectation		Interaction effect (task/expectation)		Interaction effect (task/sex)		Interaction effect (sex/expectation)		Interaction effect (task/expectation/sex)	
	F	*p*	*F*	*p*	*F*	*p*	*F*	*p*	*F*	*p*	*F*	*p*	*F*	*p*
1	3.736	0.075	3.723	0.076	1.840	0.163	0.577	0.461	0.446	0.515	0.518	0.480	0.005	0.945
2	12.860	0.001	3.738	0.065	2.199	0.110	0.879	0.487	0.140	0.936	0.121	0.731	0.089	0.890
3	2.107	0.114	3.159	0.082	1.673	0.206	0.023	0.883	0.937	0.354	0.901	0.353	0.211	0.650
4	3.091	0.060	1.585	0.218	1.371	0.257	0.239	0.628	0.837	0.584	0.197	0.661	0.820	0.375
5	2.417	0.069	1.840	0.163	1.219	0.314	0.534	0.848	0.834	0.371	0.769	0.390	0.234	0.634

6	1.006	0.411	1.290	0.305	2.388	0.137	0.110	0.741	0.997	0.400	0.188	0.669	0.547	0.468
7	2.164	0.156	0.367	0.551	3.144	0.062	0.724	0.496	0.735	0.535	0.087	0.771	0.013	0.911
8	3.226	0.059	1.857	0.143	1.878	0.185	1.099	0.366	0.133	0.718	1.196	0.329	0.103	0.752
9	4.323	0.050	3.460	0.077	1.244	0.274	1.471	0.244	0.205	0.654	0.410	0.527	0.805	0.773
10	1.301	0.267	2.386	0.133	2.499	0.129	0.512	0.480	0.426	0.519	0.547	0.468	0.184	0.671
11	2.152	0.078	2.579	0.123	3.489	0.052	1.687	0.208	1.149	0.334	1.296	0.329	0.052	0.822
12	3.306	0.061	1.624	0.212	1.733	0.202	1.249	0.297	0.762	0.525	0.026	0.873	0.768	0.391
13	3.152	0.069	2.476	0.131	2.640	0.119	0.197	0.661	0.980	0.371	0.104	0.750	0.015	0.903
14	1.858	0.186	2.663	0.118	3.675	0.060	0.066	0.799	1.994	0.121	0.455	0.507	0.413	0.528
15	0.809	0.366	1.242	0.277	1.306	0.266	0.367	0.551	0.916	0.346	0.719	0.405	0.268	0.610

Table 5

Table 5 Result of the three-factor repeated measures ANOVA in the judging stage.

Ch	Main effect of task		Main effect of sex		Main effect of expectation result		Interaction effect (task/result)		Interaction effect (task/sex)		Interaction effect (sex/result)		Interaction effect (task/result/sex)	
	F	*p*	*F*	*p*	*F*	*p*	*F*	*p*	*F*	*p*	*F*	*p*	*F*	*p*
1	0.289	0.596	3.494	0.074	3.123	0.053	0.624	0.437	0.889	0.353	0.121	0.731	2.640	0.119
2	0.006	0.938	0.032	0.860	3.482	0.051	0.929	0.345	0.021	0.885	0.001	0.974	0.820	0.375
3	0.306	0.585	2.634	0.118	8.673	0.009	2.811	0.153	0.548	0.466	0.009	0.926	0.103	0.752
4	2.079	0.162	1.371	0.253	9.184	0.002	1.663	0.209	2.231	0.148	1.622	0.215	1.112	0.302

5	0.909	0.350	0.113	0.740	3.452	0.077	1.351	0.257	1.011	0.325	0.087	0.771	0.015	0.912
6	0.656	0.426	0.286	0.598	2.525	0.069	0.988	0.330	0.272	0.607	0.188	0.669	0.009	0.926
7	0.336	0.568	0.559	0.462	3.847	0.062	1.237	0.277	3.643	0.068	2.499	0.129	2.843	0.107
8	0.500	0.486	0.245	0.625	0.538	0.470	0.024	0.879	0.418	0.524	3.675	0.069	0.085	0.773
9	1.862	0.185	0.283	0.600	0.763	0.391	3.460	0.077	0.111	0.742	0.623	0.439	1.306	0.266
10	0.021	0.885	0.519	0.478	1.196	0.285	2.231	0.148	1.622	0.215	2.388	0.137	0.104	0.750
11	0.413	0.527	1.609	0.217	0.434	0.516	0.050	0.825	0.751	0.395	0.351	0.560	0.203	0.656
12	1.527	0.228	0.019	0.893	0.624	0.437	0.156	0.696	0.769	0.396	0.188	0.669	0.367	0.551
13	3.792	0.058	0.102	0.753	0.249	0.622	0.001	0.970	1.878	0.185	0.443	0.513	0.052	0.882
14	0.008	0.930	2.820	0.106	1.285	0.268	3.450	0.056	0.026	0.873	1.733	0.202	2.579	0.123
15	3.492	0.074	0.013	0.911	3.761	0.064	3.465	0.053	1.687	0.013	0.036	0.850	0.058	0.812

Table 6

Table 6 Result of the two-factor repeated measures ANOVA between different stages

Ch	Main effect of stage		Main effect of sex		Interaction effect	
	F	*p*	*F*	*p*	*F*	*p*
1	2.218	0.124	0.036	0.991	0.594	0.619
2	9.064	0.003	0.087	0.769	1.961	0.173
3	7.268	0.011	0.833	0.480	1.247	0.317
4	2.517	0.125	0.337	0.799	0.931	0.431

5	2.107	0.127	0.436	0.515	1.253	0.298
6	4.59	0.051	1.265	0.293	8.136	0.008
7	3.450	0.060	0.100	0.755	2.029	0.138
8	2.406	0.132	0.819	0.497	0.947	0.338
9	1.181	0.287	0.431	0.731	0.402	0.752
10	11.708	<0.001	0.354	0.786	2.037	0.167
11	1.041	0.318	0.225	0.878	0.318	0.813
12	2.237	0.148	0.144	0.708	0.777	0.519
13	1.506	0.232	1.292	0.267	0.536	0.653
14	2.665	0.117	1.590	0.220	0.004	0.952
15	2.944	0.098	0.640	0.431	0.347	0.562

The red color indicates the significant value, all p values were after FDR corrected.
